# Conversion of Deoxynivalenol-3-Glucoside to Deoxynivalenol during Chinese Steamed Bread Processing

**DOI:** 10.3390/toxins12040225

**Published:** 2020-04-03

**Authors:** Huijie Zhang, Li Wu, Weixi Li, Yan Zhang, Jingmei Li, Xuexu Hu, Lijuan Sun, Wenming Du, Bujun Wang

**Affiliations:** 1 Institute of Crop Science, Chinese Academy of Agricultural Sciences, Beijing 100081, China; zhanghuijie01@caas.cn (H.Z.); wuli02@caas.cn (L.W.); liweixi@caas.cn (W.L.); zhangyan06@caas.cn (Y.Z.); lijingmei@caas.cn (J.L.); huxuexu@caas.cn (X.H.); sunlijuan@caas.cn (L.S.); duwenming@caas.cn (W.D.); 2 Laboratory of Quality & Safety Risk Assessment for Cereal Products (Beijing), Ministry of Agriculture, Beijing 100081, China

**Keywords:** deoxynivalenol-3-glucoside, deoxynivalenol, conversion, Chinese steamed bread, processing

## Abstract

We reported the conversion of deoxynivalenol-3-glucoside (D3G) to deoxynivalenol (DON) during Chinese steamed bread (CSB) processing by artificial D3G contamination. Meanwhile, the effects of enzymes in wheat flour and those produced from yeast, along with the two main components in wheat flour—wheat starch and wheat gluten—on the conversion profiles of D3G were evaluated. The results showed D3G could convert to DON during CSB processing, and the conversion began with dough making and decreased slightly after fermentation and steaming. However, there was no significant difference in three stages. When yeast was not added, or enzyme-deactivated wheat flour was used to simulate CSB process, and whether yeast was added or not, D3G conversion could be observed, and the conversion was significantly higher after dough making. Likewise, D3G converted to DON when wheat starch and wheat gluten were processed to CSB, and the conversion in wheat starch was higher.

## 1. Introduction

Trichothecenes are the most prevalent mycotoxin family, which are mainly produced by *Fusarium* species such as *F. culmorum* and *F. graminearumand*. Deoxynivalenol (DON), in particular, is the best known and predominant one due to its worldwide occurrence in cereals and products derived from them [1]. Due to the importance of wheat in diet, it is of concern that *Fusarium* can infect grains and produce mycotoxins under certain climate conditions, which can contaminate final food products. In recent years, interest has been growing in toxins called masked mycotoxins that are primarily produced in plants by enzymatic transformations related to plant resistance mechanisms to counteract pathogen invasions [2,3,4,5,6]. Masked mycotoxins, usually formed by the reaction of parent mycotoxins with amino acids or sugars, therefore, occur in conjugated forms. Furthermore, the modification may occur in the food matrix, by covalent binding or non-covalent association to sugars, proteins, or other macromolecules [7]. Deoxynivalenol-3-glucoside (D3G) is the most known masked mycotoxin, which is produced by conjugation of DON and glucose, and has been detected in various foods, such as breakfast cereals, bread, and beers [8,9,10]. The main concern about masked mycotoxins is that their conjugates can be hydrolyzed in the stomachs of mammals after uptake, thereby releasing the toxic precursor DON and influencing its bioaccessibility [11]. The Joint FAO/WHO Expert Committee on Food Additives (JECFA) declared that D3G could contribute to DON dietary exposure; research on its absorption, distribution, metabolism, excretion in animal and human body, and its fate in food processing are needed [12]. The acetylated derivatives of DON, 15-acetyl-deoxynivalenol (15-AcDON) and 3-acetyl-deoxynivalenol (3-AcDON), are intermediate products of fungal DON biosynthesis that generally occur together with DON in cereal commodities, and 3-AcDON can convert to DON during mammalian metabolic processes and thus contribute to the total DON toxicity [13]. Therefore, AcDONs are considered to be masked mycotoxins by some researchers [7], and the JECFA amended the provisional maximum tolerable daily intake (PMTDI) for DON to 1 mg/kg bodyweight for DON and its acetylated forms [12].

DON’s glucoside form has been found in various raw cereals. It was reported that the mean concentrations of D3G were 393 μg/kg and 141 μg/kg, respectively, for wheat and maize samples [14]. Palacios et al. (2017) [15] reported that D3G was detected in 94% of the investigated durum wheat commercial cultivars from the Argentinean main growing area at concentrations ranging from < the limit of quantification (LOQ, 50 μg/kg) to 850 μg/kg. Lancova et al. (2008) [16] found D3G in naturally contaminated barley, and a remarkable increase of D3G was observed in malt compared to raw barley. Ksieniewicz-Wo’zniak et al. (2019) [17] investigated D3G levels in 87 barley malt samples, and 91% were positive, with concentrations ranging from 4.4–410.3 µg/kg. Quite a lot of similar reports have been presented [18,19,20].

Previous research indicates that mycotoxins may transform in food processing via heating, fermentation, or from ingredients such as enzymes [21]. It was also reported that food processes, such as sorting, cleaning, milling, brewing, baking, frying, roasting, alkaline cooking, extrusion, etc., might affect mycotoxins [22]. Many agricultural products that contaminated with mycotoxins are processed using germination (barley, for example), fermentation, hydrolysis, enzymes, and alkaline or acidic hydrolytic conditions, which contribute to the production or release of masked mycotoxins [23]. D3G can be transformed to DON in food processing, and vice versa [24]. The cleavage of masked mycotoxins occurs during the process of malting, leading to an increase in free DON [8]. DON contents increased after baking, which suggests that bound DON is released in baking [25].

Among the many factors influencing masked mycotoxin transformation during food processing, enzymes produced from microorganisms have garnered substantial interest from researchers worldwide. It was reported that doughnuts fermented with yeast contaminated higher DON content than that in the flour, and this may be due to enzymatic transformation [26]. Simsek et al. (2012) [25] reported the effects of milling and baking processing on D3G. The addition of enzyme mixtures to improve baking increased the content of D3G in fermented dough up to 145%. D3G and DON levels decreased slightly in baking. When whole wheat was treated with enzymes to evaluate the effects of enzymatic hydrolysis on DON, the results showed that DON contents were prominently higher after treatment with protease (16%) and xylanase (39%), which suggested DON maybe embed or bind in the cell wall or protein component of wheat kernel. Bread processing usually involves fermentation with yeast or leavening agents (leaven). In particular, rye bread with leaven requires more extensive enzymatic activity [27].

In our previous study, we found that the DON levels in baked bread were almost double those in flour, while the D3G contents in baked bread were notably lower. The increase in DON and the decrease in D3G started during the fermentation of the dough. DON contents approximately doubled after mixed and fermented dough was processed to CSB, and D3G concentrations were almost 50% lower than in flour, suggesting that CSB processing may release bound DON in the flour [28,29]. Our research team investigated the fate of 3-ADON and 15-ADON in bread processing by spiking mycotoxin-free wheat flour with 3-ADON and 15-ADON standards, and the results showed that ADONs could convert to DON during bread processing [30].

The objective of the assay presented in this study was to investigate whether D3G could convert to DON during CSB processing, as suggested by our previous research. The effects of enzymatic hydrolysis and different wheat compositions on D3G conversion were verified.

## 2. Results

### 2.1. Conversion of D3G during CSB Processing

In our previous study, wheat flour naturally contaminated with DON and D3G was used for wheat-based product processing. Thus, it was generally contaminated with small amounts of other derivatives of DON, especially other masked DONs. To elucidate the behavior of D3G during Chinese steamed bread processing, D3G contaminated wheat flour samples were prepared in this study by spiking wheat flour, free of target mycotoxins, with a standard solution of D3G.

DON concentrations in the doughs (mixed dough and fermented dough) and steamed products produced from wheat flour spiked with three different D3G levels are presented in Table 1. DON was detected in the whole processing of CSB, and the amount of DON converted from D3G was proportional to the spiked amount. In addition, the DON concentrations converted from D3G during the whole processing decreased, and there was no significant difference (*p* < 0.05) among mixed dough, fermented dough, and steamed products. The results indicated that D3G could release DON in CSB processing and that the conversion started with the dough making process. The results agreed with those from our previous research, in which D3G concentration changes started with the dough making process [28].

### 2.2. Role of Enzymes in the Conversion of D3G during CSB Processing

**Figure 1:**
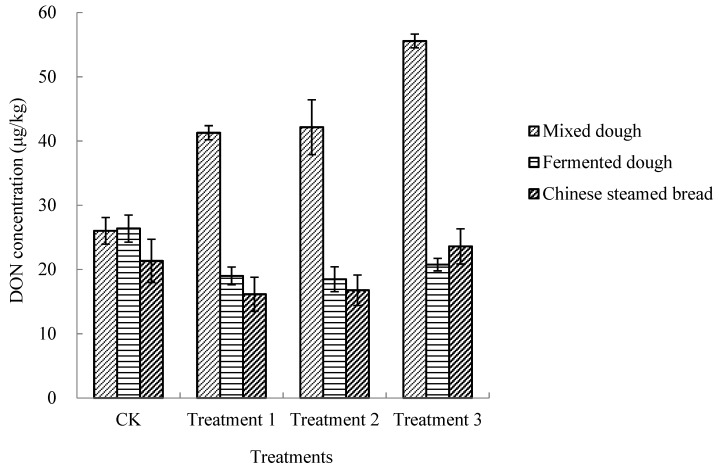
DON concentrations converted from spiked D3G (300 μg/kg) in different treatments of Chinese steamed bread. CK: wheat flour, yeast used; Treatment 1: wheat flour, no yeast used; Treatment 2: enzyme-deactivated wheat flour, yeast used; Treatment 3: enzyme-deactivated wheat flour, no yeast used.

Previous research suggests that D3G can release DON during food processing as a result of enzymatic degradation of polysaccharides [25,26]. Yeast and wheat flour were two sources of enzymes in CSB processing. Wheat flour contains several important enzymes, such as amylases, proteases, lipoxygenase, polyphenol oxidase, and peroxidase. These enzymes are inactive during grain and flour storage, and they become active when water is added [31]. Enzymes produced by yeast also play an important role in dough fermentation. The results of the present study showed that D3G could convert to DON during CSB processing without using yeast (Treatment 1) and when using enzyme-deactivated wheat flour with the addition of yeast (Treatment 2). The conversion started from dough making, as observed in the CSB processing of wheat flour with added yeast (CK) (Figure 1). In addition, the conversion was also observed when enzyme-deactivated wheat flour was processed to CSB, and with no yeast used (Treatment 3). While DON concentrations converted from D3G after dough making in these three treatments were significantly higher than that of the control, they decreased dramatically after dough fermentation and decreased or increased slightly after steaming. Except when enzyme-deactivated wheat flour with no yeast was used in CSB processing, the DON levels in fermented dough and steamed products were significantly lower than those of the control. These results suggested that D3G could convert to DON during CSB processing in the absence of yeast or enzymes in wheat flour or both of them, while the production of DON in steamed products was generally lower. The significant increase in the concentration of DON after dough making merits further research.

### 2.3. Conversion of D3G in Different Wheat Compositions during CSB Processing

**Figure 2:**
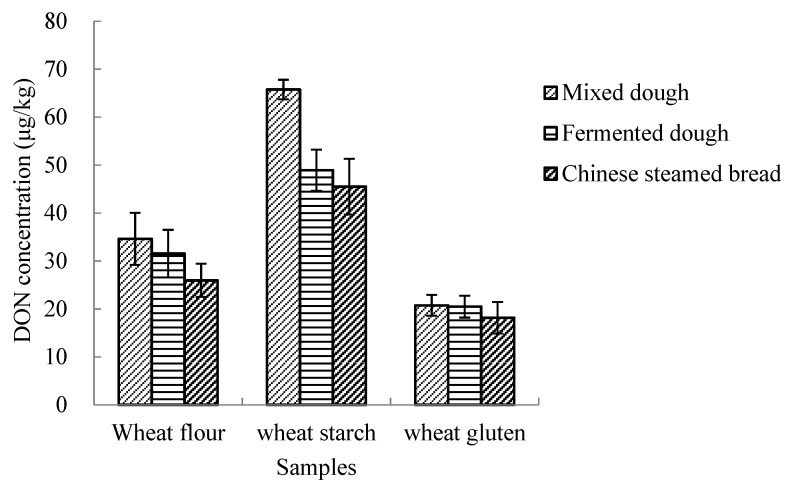
DON concentrations converted from spiked D3G (500 μg/kg) during Chinese steamed bread processing with different wheat compositions.

The fate of mycotoxins in food processing was affected by several factors, such as the food matrix, pH, moisture content, temperature, natural or spiked contamination, and original mycotoxin concentration [32]. To determine whether the compositions in flour play an important role in D3G conversion during CSB processing, wheat starch and wheat gluten were chosen to simulate CSB processing, since they account for approximately 75% and 10%, respectively, of wheat flour. The results showed that D3G conversion also occurred during the CSB processing of wheat starch and wheat gluten (Figure 2). The conversion of D3G in wheat starch was significantly higher than that of wheat flour and wheat gluten in dough mixing, fermentation, or steaming, and the conversion amounts of D3G in wheat gluten was the lowest. As observed in the CSB processing of three wheat flour samples spiked with three different D3G levels, the DON conversion from D3G during the CSB processing of wheat starch and wheat gluten decreased. No data are available on the conversion of D3G in different wheat compositions during CSB processing. The results of this study suggest that amylose or amylopectin in wheat flour may play a crucial role in D3G conversion during CSB processing, and that this mechanism merits further study.

## 3. Discussion

This study confirmed that D3G could convert to DON during CSB processing, and the conversion started from the dough making process and decreased slightly after dough fermentation and steaming. In addition, D3G could convert to DON during CSB processing in the absence of yeast or enzymes in wheat flour or both of these components, while converted DON in steamed products was generally lower. In addition, D3G conversion could occur when wheat starch and wheat gluten were processed to CSB, and the amounts converted in wheat starch were significantly higher.

The results of this work indicated that the conversion of D3G in CSB processing began with dough mixing. Our previous research indicated that DON levels increased significantly after dough was rolled in a noodle machine during noodle processing, and that D3G levels decreased dramatically [29]. These studies suggested that mechanical force may exert a remarkable influence on mycotoxin structure, therefore leading to the liberation of masked mycotoxins. To verify this hypothesis, we studied the effect of mechanical forces (oscillate in an ultrasonic cleaner and blend at high speed in a blender for 30 min, respectively) on the conversion of D3G by spiking D3G standard in water and acetonitrile. The results (data not listed) showed that only trace amounts of D3G (approximately 1%) converted to DON after oscillating or blending. Further research indicated 15-AcDON and 3-AcDON could also convert to their parent mycotoxins when 15-AcDON and 3-AcDON standard spiked in water or acetonitrile were oscillated in an ultrasonic cleaner, blended at high speed in a blender, shaken in a shaking incubator, or stirred in a magnetic stirring stirrer; likewise, only a little bit of DON could be detected after treating. The results suggested that mechanical force had an impact on the structures of D3G, 15-AcDON, and 3-AcDON, leading to the conversion to DON, and the conversion of D3G standard (in acetonitrile) under mechanical forces indicated that the hydroxyl of water molecules was not a crucial factor in D3G conversion. There is no description of this phenomenon in the existing literature. This may lead us to a new realm called mechanochemistry, which involves mechanical and chemical behaviors on a molecular level, and various phenomena, such as mechanical breakage, polymeride degradation by shearing, cavitation-involved phenomena, shock wave chemistry and physics, molecular machines, etc., are included. Mechanochemistry could be regarded as the intersection of mechanical engineering and chemistry, and it produces chemical product synthesis depending on possible mechanical actions. The mechanisms of mechanochemical transformations are different from those of general techniques [33]. Ball milling is a widely used technology, in which chemical processing and transformations are moved by mechanical force [34]. In this study, the generation of DON under mechanical forces may occur due to the mechanical energy of mechanical friction and shear exerted upon mycotoxin molecules that lead to structural changes of D3G, 15-AcDON, and 3-AcDON. However, as only a trace amount of D3G could convert to DON in liquid under mechanical forces, and CSB processing generally involves different compositions in a flour matrix, ingredients and complex physico-chemical modifications that occur in the processing, D3G conversion in CSB processing may be due to other mechanisms, and it still needs further research.

## 4. Materials and Methods

### 4.1. Chemicals and Reagents

The analytical standard of D3G (50 μg/mL in acetonitrile, certified purity >99.9%) was purchased from Sigma (Sigma-Aldrich, Alcobendas, Spain). Purified water was obtained from a Milli-Q apparatus (Millipore Corp., Bedford, MA, USA). Methanol, acetonitrile and formic acid (all HPLC grade) were purchased from Thermo Fisher Scientific Corporation (Shanghai, China).

### 4.2. Preparation of D3G-Contaminated Wheat Flour/Enzyme-Deactivated Wheat Flour/Wheat Starch/Wheat Gluten

In the present experiment, wheat flour, wheat starch and wheat gluten samples with undetected levels of target mycotoxins were designated to be “blank”. Wheat flour was purchased from Huanghua Jinmai Flour Co., Ltd. (Cangzhou, Hebei, China). Wheat starch was purchased from Shanghai Saiwengfu Agricultural Development Co., Ltd., and wheat gluten was from Shandong Qufeng Food Tech Co., Ltd. Blank wheat flour was dried at 130 °C in a drying oven (DHG-9140A, Shanghai Yiheng Scientific Instruments Co., Ltd., Shanghai, China) for three hours to prepare enzyme-deactivated blank wheat flour. D3G-contaminated wheat flour/enzyme-deactivated wheat flour/wheat starch/wheat gluten samples were prepared by spiking blank samples with a standard solution of D3G.

### 4.3. Preparation of Chinese Steamed Bread

Chinese steamed bread was processed according to the Chinese Business Standard (procedure 10139-93, Appendix A, 1993) with some modifications. The experiment was conceived and executed as follows: (1) blank wheat flour spiked with 3 levels of 300, 500, and 800 μg/kg D3G (based on the dry sample as delineated below), mixed with yeast solution (0.26 g dry yeast dispersed in 12.5 mL water at 38 °C, as delineated below); (2) blank wheat flour spiked with 300 μg/kg D3G, mixed with 12.5 mL water (38 °C) instead of yeast solution; (3) enzyme-deactivated blank wheat flour spiked with 300 μg/kg D3G, mixed with 12.5 mL yeast solution, and an additional 12.5 mL water (38 °C) was added to form the resultant dough; (4) enzyme-deactivated blank wheat flour spiked with 300 μg/kg D3G, mixed with 25 mL water (38 °C) instead of yeast solution; (5) blank wheat starch spiked with 500 μg/kg D3G, mixed with 12.5 mL yeast solution; (6) blank wheat gluten spiked with 500 μg/kg D3G, mixed with 12.5 mL yeast solution and additional 7.5 mL water (38 °C). Each treatment was prepared in triplicate. After 3 min of mixing, the mixed doughs were fermented in a fermentation cabinet (38 °C, 85% RH) for 60 min. Then manually molding to produce a round dough with a smooth surface was performed for 3 min, followed by steaming at 100 °C in a steaming chamber for 20 min after putting in air for 15 min. Then the steamed dough was cooled at room temperature for 40–60 min. Sampling was conducted at the end of dough preparation, after fermentation, and after steaming. Representative subsamples were stored at −20 °C until analysis.

### 4.4. Sample Treatment and UPLC-MS/MS Analysis

Sample treatment was performed as described by Zhang and Wang (2014) [28] with slight modifications. Ten mL acetonitrile: water (80:20, *v/v*) was blended with two grams of a representative sample and extracted for 3 min with a blender (IKA Co., Staufen, Germany). Then the sample was centrifuged at 10,000 rpm for 10 min, and the supernatant (5 mL) was filtered through a multifunctional MycoSep 226 columns (Romer Labs, Inc. Union, MO, USA), and 2 mL of the extract was submitted to N-EVAP at 50 °C until dry. Sequentially, the residue was dissolved in a mixture of 0.4 mL methanol: water (50:50, *v/v*), then vortexed and filtered through 0.22 μm MICRO PES filter (Membrana, Germany) for UPLC-MS/MS analysis.

The UPLC-MS/MS analysis of DON was conducted as described by Zhang and Wang (2014) [28] with some modifications. A multiple reaction monitoring (MRM) mode was performed, and column temperature was set at 26 °C, capillary voltage at 2.5 KV, cone voltage at 20 V. Gaseous nitrogen was used as desolvation gas, and its flows were maintained at 800 L/h. Desolvation temperature was set at 450 °C. Mobile phase A was methanol and mobile phase B was 0.1% (*v/v*) formic acid in water. Gradient of phase A performed was 0.2 mL/min for 0–3.5 min, and increased linearly from 5% to 85%, for 3.5–4.5 min a linear increase from 85% to 100% was followed, then decreased from 100% to 5% for 4.5–5.0 min, and followed by an isocratic washout of 5% A for 1 min.

## Figures and Tables

**Table 1:** Deoxynivalenol (DON) concentrations converted from spiked deoxynivalenol-3-glucoside (D3G) at different stages of Chinese steamed bread processing.

Samples	Spiking Levels (μg/kg)		
	300	500	800
Mixed dough	26.02 ± 2.07a	34.64 ± 5.42a	61.75 ± 4.88a
Fermented dough	26.37 ± 2.11a	31.55 ± 4.96a	54.42 ± 5.16a
Chinese steamed bread	21.33 ± 3.37a	25.96 ± 3.46a	52.16 ± 5.69a

DON concentrations were average values based on three replicates. Means followed by the same small letters within columns are not significantly different (*p* > 0.05).
